# Effects of age, size, and mating history on sex role decision of a simultaneous hermaphrodite

**DOI:** 10.1093/beheco/aru184

**Published:** 2014-10-17

**Authors:** Yumi Nakadera, Elferra M. Swart, Jeroen P.A. Maas, Kora Montagne-Wajer, Andries Ter Maat, Joris M. Koene

**Affiliations:** ^a^Section Animal Ecology, Department of Ecological Science, Faculty of Earth and Life Sciences, VU University Amsterdam, De Boelelaan 1085, 1081HV Amsterdam, The Netherlands,; ^b^Applied Biology, HAS University of Applied Sciences, Onderwijsboulevard 221, 5223DE ‘s-Hertogenbosch, The Netherlands, and; ^c^Behavioural Neurobiology, Max Planck Institute for Ornithology, Eberhard-Gwinner-Strasse, 82319 Seewiesen, Germany

**Keywords:** *Lymnaea stagnalis*, reproductive strategy, sex allocation, sexual conflict, size-advantage model.

## Abstract

Contrasting with separate-sexed animals, simultaneous hermaphrodites display unique reproductive strategies as they are male and female at the same time. Simultaneous hermaphrodites that copulate unilaterally, for instance, make a decision to mate as a male or female. Previous studies have demonstrated that sex role preference in hermaphrodites is flexible and is controlled by several, often confounding, factors. We examined the relationship between sex role decisions and 3 life-history traits (age, size, and mating history) in the great pond snail, *Lymnaea stagnalis*. Based on our field observations, which indicate that adult individuals show overlapping generations and large variation in body size during the breeding season, we performed a sex role choice experiment in the laboratory. We found that young and small snails mate as males first. Both age and size significantly affected sex role decision, with age having a stronger effect. Furthermore, we tested whether *L. stagnalis* becomes reluctant to inseminate a mate after being inseminated because it is known that after insemination, male investment substantially reduces. Contrary to expectations, our results indicate that the receipt of seminal fluid does not seem to reduce male motivation. In sum, sex role decisions in *L. stagnalis* are largely determined by age and size but not by having received seminal fluid. This mating pattern, however, does not fully support the size-advantage model because large or old individuals did not perform better as females in our experiment. These results imply a conflicting mating interest, rather than harmonious agreement, between age- and size-different hermaphrodites.

## Lay summary

Quite a few animals are male and female at the same time, so they can choose to mate either as male or female on copulation. The decision to perform either sex role was known to be highly flexible depending on various, but often confounding, factors. For the pond snail, we report that young and small snails tend to mate as males first, though old and large snails do not seem to be better females.

## INTRODUCTION

Simultaneous hermaphrodites (hereafter, hermaphrodites) can have several reproductive strategies that separate sexes do not possess. For instance, on copulation, they need to decide whether to mate as a male or female (sex role decision), which is predetermined in separate-sexed animals (Anthes et al. 2006a). Although the decision to mate in one sex role is restricted to hermaphrodites with unilateral copulation (i.e., mating as either a male or a female in a single insemination event), the same considerations can apply to reciprocal copulation, though such hermaphrodites mate as male and female at the same time. In the latter, though, the endpoint of this decision is the resource allocation to male or female functions (Baur et al. 1998). Thus, hermaphrodites, with their availability of sex role options, provide a unique arena for having precopulatory reproductive strategies that allow them to flexibly change sex roles depending on their immediate circumstances.

In hermaphrodites, sex role decisions seem highly flexible and condition dependent (Anthes et al. 2006a; Table 1). It has often been assumed that hermaphrodites have a fixed male or female role preference, preferring the one with the least expenditure (Leonard and Lukowiak 1984; Leonard 1991; Michiels et al. 2003). However, recent theoretical and empirical studies demonstrated that patterns of sex role decisions are highly flexible, depend on multiple cues, and vary between closely related species (Anthes et al. 2006a; Anthes 2010; summary of pulmonates in Table 1). This flexibility in sex role decisions could be an important consequence of sexual selection, analogous to the plastic female mate preference in separate-sex species (e.g., Hunt et al. 2005; Chaine and Lyon 2008; Tinghitella et al. 2013). Thus, previous studies have unveiled a great plasticity and vast complexity in sex role decisions in hermaphrodites, though the underlying mechanism still needs elucidation.

**Table 1:** A review of factors influencing sex role decisions in pulmonates

Factor	Sex role decision	Species	Origin	Reference
Body size	Small snails donate	*Physa gyrina*, *Physa heterostropha*	Wild, laboratory	DeWitt (1996)
	Small snails donate	*Helisoma trivolvis*	Laboratory	Norton et al. (2008)
	Small snails donate?	*Bulinus africanus*	Laboratory	Rudolph and Bailey (1985)
	NS	*Cepaea nemoralis*	Wild	Wolda (1963)
	NS	*Lymnaea stagnalis*	Laboratory	Koene et al. (2007)
	Small snails donate	*Physa acuta*	Wild	Ohbayashi-Hodoki et al. (2004)
	Donate to large snails	*Radix lagotis*	Wild	Yu and Wang (2013)
	NS, donate to large snails	*Succinea putris*	Wild	Jordaens et al. (2005) and Dillen et al. (2008, 2010)
	NS	*Siphonaria capensis*	Wild	Pal et al. (2006)
	NS	*Arianta arbustorum*	Wild	Baur (1992) and Baur et al. (1998)
	Size assortative mating?	*Helix pomatia*	Wild	Baur (1992)
Mating history	Isolated snails donate	*L. stagnalis*	Laboratory	Van Duivenboden and Ter Maat (1985) and Koene and Ter Maat (2005)
	NS (virgin or nonvirgin)	*L. stagnalis*	Laboratory	Koene et al. (2008)
	Isolated snails donate	*P. heterostropha pomilia*	Lab	Wethington and Dillon (1996)
	Isolated snails receive	*P. acuta*	Laboratory	Facon et al. (2007)
	Isolated snails donate	*P. gyrina*	Wild	McCarthy (2004)
	Isolated snails donate	*S. putris*	Wild	Dillen et al. (2008)
	Virgins donate/receive more	*Euhadra quaesita*	Laboratory	Kimura and Chiba (2013)
	NS (virgin or nonvirgin)	*A. arbustorum*	Wild	Baur et al. (1998)
Genetic background^a^	Avoid allopatric snails	*Biomphalaria glabrata, Biomphalaria pfeifferi*	Laboratory	Rupp and Woolhouse (1999)
	Avoid sympatric or allopatric snails	*P. gyrina*	Wild, laboratory	McCarthy (2004) and McCarthy and Sih (2008)
	Avoid sympatric snails	*P. acuta*	Laboratory	Facon et al. (2006)
	Avoid sympatric or allopatric snails	*A. arbustorum*	Wild	Baur and Baur (1992)
	NS (full-sib or nonsib)	*A. arbustorum*	Laboratory	Baur and Baur (1997)
	Avoid sympatric or allopatric snails	*Cornu aspersum*	Wild	Fearnley (1996)
Morphology	Donate to same coiling direction	*Partula suturalis*	Wild, laboratory	Johnson (1982)
	Donate to same coiling direction	*Bradybaena similaris*	Laboratory	Asami et al. (1998)
	Donate to same coiling direction	*L. stagnalis*	Laboratory	Koene and Cosijn (2012)
	Donate to opposite coiling direction	*Amphidromus inversus*	Wild	Schilthuizen et al. (2007)
Mate identity	Donate to new mates	*L. stagnalis*	Laboratory	Koene and Ter Maat (2007)
	NS (new or familiar)	*B. glabrata*	Laboratory	Häderer et al. (2009)
Age	Young snails donate	*L. stagnalis*	Laboratory	Hermann et al. (2009)
	Donate to old snails	*Achatina fulica*	Wild	Tomiyama (1996)
Infection state	Avoid infected snails	*B. glabrata*	Laboratory	Webster et al. (2003)
Shell color, band pattern	NS	*C. nemoralis*	Wild	Wolda (1963)
Dart shooting	NS (sperm transfer)	*C. aspersum*	Wild	Chase and Vaga (2006)

NS = no significant pattern observed; ? = weak trend with or without statistical test.

^a^Difference in genetic background is usually assumed due to geographic distance of original populations and low dispersal ability of pulmonates.

We propose 2 approaches to further understand sex role decisions in hermaphrodites. First, an explicit experimental design is required to evaluate the relationship between sex role decision and candidate factors and traits because most of the identified ones are confounded (Table 1). Body size, which is the best documented trait, strongly correlates with age in animals with indeterminate growth (Jordaens et al. 2005; Hermann et al. 2009; Table 1). However, when one tests the effect of size on sex role decision, the influence of age is usually not controlled for or even described (11 out of 12 cases, except for Rudolph and Bailey 1985). In previous studies, either the effect of body size on sex role decision was specifically excluded by size matching (Hermann et al. 2009) or the age was unknown because animals were caught from the wild (Monroy et al. 2005; Anthes et al. 2006b; Sprenger et al. 2009; Table 1). Hence, we have designed an experiment to separately evaluate the effects of age and body size on sex role decisions in hermaphrodites.

Second, it is necessary to integrate the knowledge of pre- and postcopulatory processes in order to predict a potential decision-making process on sex role. Despite the ability to self-fertilize in many hermaphrodites, they mate repeatedly and polyandrously (Baur 1998; Michiels 1998), suggesting a significant role for postcopulatory processes in sexual selection (Nakadera and Koene 2013; Schärer and Pen 2013). Moreover, the impact of postcopulatory processes could interact with precopulatory processes because both levels of sexual selection are expected to coevolve for optimal reproduction (Fromhage et al. 2008; Parker et al. 2013). This means that hermaphrodites could reach their precopulatory decision based on recent postcopulatory events. For instance, the great pond snail, *Lymnaea stagnalis* (L.), nearly halves the number of sperm transferred in a subsequent male mating when they recently received seminal fluid proteins (Nakadera et al. 2014). Given such a postcopulatory change in this species, one would anticipate snails to be reluctant to mate as males after being recently inseminated because their expected male reproductive performance is reduced (Nakadera et al. 2014).

Here, we examined the effect of age and size on the sex role decision in *L. stagnalis* by adopting a two-by-two factorial experimental setup. First, we estimated natural variation in age and size in a field population. Then, including age and size as independent factors within the ranges found in the wild, we carried out a laboratory experiment to pair large or small, young or old snails to observe which ones copulate as a male first. Given the resulting mating pattern, that young or small snails tend to perform the male role first, we examined whether old or large snails are more fecund or allocate less to their male function. In addition, we tested whether they become reluctant to mate as a male after being inseminated, either due to the reduced postcopulatory male performance that they experience or due to the action of a seminal fluid protein that specifically targets male motivation.

## MATERIALS AND METHODS

### Field survey

We collected a total of 564 *L. stagnalis* (Lymnaeidae, Panpulmonata) from the ditches in Eempolder near Amsterdam, the Netherlands, over 2 years (see also Ter Maat et al. 2007). Samples were collected monthly using a sweep net to obtain a representative subset of the population. The shell lengths of all individuals were measured on collection using calipers. In the laboratory, we dissected the snails to determine the state of parasitic infection, which is known to affect both body size (e.g., gigantism) and reproductive ability (castration; McClelland and Bourns 1969; Sorensen and Minchella 2001). On average, 50% of the snails collected were infected with one or more parasitic species, and infection rate varied seasonally (Loy and Haas 2001; Żbikowska and Żbikowski 2005; Ter Maat et al. 2007; see Results). All infected individuals and the juveniles (being too small to diagnose confidently, shell length < 18mm) were removed from the analyses, giving us a sample size of 281 adults. Based on these data, we estimated age cohorts and growth rate in the wild (see Statistics).

In addition, we measured water temperature in the ditches during sampling and noted the photoperiod because both these factors have been shown to affect the reproduction of *L. stagnalis* (e.g., Dogterom et al. 1984; Ter Maat et al. 2007, 2012). Also, we recorded reproductive activity, that is, presence of egg masses and pairs that were copulating. Although we could not measure all the copulating pairs seen in the field, observed copulations included pairs with large size differences. This is consistent with previous work showing that spontaneously copulating individuals, ranging between 18 and 31mm, can and do mate with each other (Koene et al. 2007).

### Sex role choice experiment

We used a laboratory culture of *L. stagnalis*, which has been maintained at VU University Amsterdam for about 50 years. The original collection site of this culture is the same area where we did our field survey. In the flow-through tanks, snails are kept in aerated, low-copper water at 20±1 °C, with a light:dark cycle of 12:12h. They are fed broadleaf lettuce and fish flakes (Tetraphyll, Tetra GmbH) ad libitum. Several age-synchronized cohorts (hatched from egg masses laid within 24h), ranging from eggs to 4 months old, are bred continuously.

To test their sex role decision, we allowed pairs of snails to mate under observation. Freshwater snails copulate unilaterally, whereby one of the individuals acts in the male role, whereas the other acts in the female role. After showing stereotypical precopulatory behavior (Jarne et al. 2010; Koene 2010), the male-acting individual (donor) inseminates its partner (recipient). In short, the donor mounts the recipient’s shell, positions itself, and eventually everts its penis-carrying organ, the preputium, to ultimately reach intromission into the female genital pore (gonopore). Insemination takes 20–60min. The frequency of mating is relatively high as snails can donate ejaculates twice per day after a week of isolation (Koene and Ter Maat 2007). The mating characteristics of this species allow us to readily discriminate which individual mates in the male role first (primary donor) in any pair of snails.

We investigated whether age or size determines the primary donor by adopting a two-by-two factorial design. From our mass culture, we obtained small and large snails with a 5 mm difference in shell length (mean ± standard error [SE]: small = 24.84±0.07mm, *N* = 84; large = 29.54±0.08mm, *N* = 84) from 2 different age cohorts (3- and 4-month olds from egg-laying date). Although their life span can reach over a year, for simplicity we called the age-different snails Young and Old (e.g., Janse et al. 1989; Hermann et al. 2009). Thus, this collection gave us 4 groups: Young/Small, Young/Large, Old/Small, Old/Large. Shell length strongly correlates with body mass, making it a suitable measure for body size (Zonneveld and Kooijman 1989; Koene et al. 2008). In addition, it has been shown that a difference in shell length of 5mm does not physically inhibit mating (Koene et al. 2007). Although this species first matures as male (slight protandry, Jordaens et al. 2007), 3-month-old snails are already fully hermaphroditic, which is indicated by egg-laying capabilities (Van Duivenboden 1983; Koene et al. 2008). As most freshwater snail species, *L. stagnalis* shows indeterminate growth. Crucially, at this stage (3- to 4-month old), they undergo a rapid growth phase, giving rise to large variance in body size within each age-synchronized cohort (Zonneveld and Kooijman 1989; Koene et al. 2008; Hermann et al. 2009). Such variation within both age cohorts allowed us to find suitable individuals differing only in size.

To measure which snail assumes the male role first, we assigned the 4 groups to 2 treatments: same age but different sizes (Young/Small × Young/Large pair, *N* = 21; Old/Small × Old/Large pair, *N* = 21) or similar size but different ages (Young/Small × Old/Small pair, *N* = 21; Young/Large × Old/Large pair, *N* = 21). These 4 combinations were selected as they allowed us to isolate the effect of age and size on sex role decisions. Hence, combinations where both traits were different (i.e., Young/Small × Old/Large, Young/Large × Old/Small) were excluded, though they might represent the interactive effect of age and size. Combinations of snails with same age and size were also excluded because they cannot provide a comparable measurement (i.e., whether young or old, large or small snail is the primary donor).

In order to standardize male mating motivation, we isolated the snails in perforated containers within a large flow-through tank. During 8 days of isolation, the snails fully replenish the seminal fluid in their prostate glands (De Boer et al. 1997), thereby enhancing male motivation (Van Duivenboden and Ter Maat 1985; Hermann et al. 2009). During isolation, each snail was fed 1 lettuce disc (≈19.6cm^2^) per day, which is slightly below its maximum intake (Zonneveld and Kooijman 1989). The isolation period also allowed us to observe if the snails were fully matured (hermaphroditic) by examining their egg-laying capabilities. All nonlaying individuals were excluded from the experiment.

After isolation, we paired the snails and observed their mating behavior. We put preassigned snails together into a closed container (500mL) filled with low-copper water at room temperature. For identification, we marked one of the snails in the pair with a small dot of nail polish. During observation, we recorded if there was no contact or whether one of them was mounting, probing (here called “courtship behavior”), or inseminating (attachment of the preputium onto the gonopore), every 15min for 6h or until the completion of reciprocal mating (both snails mated as male and female). The observation time is long enough for a pair of snails to reciprocate insemination.

### Effect of age and size on female reproductive output and sex allocation

We carried out an independent observational study to test the female reproductive output between the different ages and sizes. In total, we isolated small and large snails for 11 days from 2 different age cohorts (3.5- and 4.5-month-old). Four days of isolation were necessary to allow the physiological effects of previous inseminations to disappear (Koene et al. 2009, 2010; Hoffer et al. 2010) and 7 days to collect new egg masses laid. The egg masses were scanned under glass plates on a flatbed scanner (Canoscan LiDE 700F, Canon) at ×150 magnification, including an eyepiece micrometer as scale. From these scanned images, we determined egg number and size (ImageJ; NIH, Wayne Rasband). For egg size, we measured the area (mm^2^) of 5 randomly selected eggs per egg mass. Egg masses were then freeze-dried to determine their dry weights.

Sex allocation was estimated by measuring the dry weights of male and female reproductive organs. On day 11 after measuring their shell lengths, they were sacrificed by injecting about 3mL of 50mM MgCl_2_ through their foot. We then dissected out their prostate glands (producing seminal fluid, male organ), seminal vesicles (storing autosperm, male organ), and albumen glands (producing albumin for eggs, female organ). We freeze-dried the organs and the remaining soft body parts to measure their dry weights. Given the function of the albumen gland, we also noted whether the snails had laid egg masses within the last 24h. At the end of this experiment, shell lengths were as follows (mean ± SE): Young/Small, 28.18±0.21mm (*N* = 19); Young/Large, 32.16±0.16mm (*N* = 20); Old/Small, 28.20±0.19mm (*N* = 13); and Old/Large, 31.43±0.18mm (*N* = 17).

### Postcopulatory male reluctance experiment

We tested whether being recently inseminated affected male mating motivation. First, we isolated 38 snails for a week to ensure high male mating motivation and assigned them as inseminators (Van Duivenboden and Ter Maat 1985; De Boer et al. 1997; Hermann et al. 2009). Next, focal and recipient snails (*N* = 76) were isolated for 4 days, just long enough to remove any physiological effects of previous matings (Koene et al. 2009, 2010; Hoffer et al. 2010). We randomly assigned these snails according to body size, ensuring no size difference between these 3 snail types (mean ± SE: inseminators = 31.27±0.27mm; focals = 30.81±0.29mm; recipients = 30.67±0.25mm; Anova, *F*_2,99_ = 0.643, *P* = 0.528) or within each pair (differences of focals and recipients between treatments, *t*-test, *t*_26.32_ = 0.424, *P* = 0.675; differences between inseminators–focals and focals–recipients, *t*_19.42_ = 0.762, *P* = 0.455). For identification, the shells of the focal snails were marked with a small dot of nail polish.

Half the focals were paired to inseminators, thereby becoming inseminated donors (*N* = 38), while their mating behavior was monitored by following the procedure above. During observation, the focals in the control group were placed in closed containers without a mating partner. The inseminators successfully inseminated the focal snails in 19 of the 38 pairs, but four of them were discarded due to handling error. After measuring shell lengths, the 15 inseminated and 30 control focals were isolated in perforated containers again. The following day, in closed observation containers, focals were randomly paired to a recipient snail under observation (control pairs, *N* = 30; inseminated focal pairs, *N* = 15). Note that all the snails were equally motivated to mate as males, and the only difference between the 2 groups was the fact that the inseminated focal snails received one ejaculate the day before.

### Statistics

To examine their overlapping generations in terms of different age cohorts in the field, we performed a K-means clustering, partitioning the observations into 2 clusters, based on date of collection and shell length. An observation is assigned to a cluster with the nearest mean, thereby the smallest Euclidean distance. Diagnostic checks allowing for more than 2 clusters did not yield meaningful results. For each of the 2 clusters, we fitted a Von Bertalanffy growth curve (Zonneveld and Kooijman 1989; Ter Maat et al. 2007; Koene et al. 2008), relating size increase to equal time intervals between age groups by the method of least squares. These estimates allowed us to examine whether these 2 clusters show a similar growth pattern, to provide support for distinct age cohorts in the wild.

In the sex role choice experiment, to ensure that each snail within a pair was capable of insemination, we excluded all pairs where one or both snails did not mate as a male (see Results). Our data set, therefore, comprised reciprocating pairs, that is, both individuals having mated as a male and a female. We registered whether or not a young or small snail mated as the primary donor (yes/no) within age- and size-different treatment, respectively (e.g., in the age-different data set, 1 means young snails inseminate first and 0 means it was inseminated first). These 2 data sets were entered into binomial tests in order to examine whether young or small snails tend to play male role first. Then, we used a generalized linear model (GLM) with binomial distribution together with logit link function, giving size and age as fixed factors to age- and size-different treatment, respectively. This procedure allows us to test whether the first male tendency differed between the 2 groups. In addition, we tested whether mating behavior (courtship and insemination duration) differed between ages and sizes. We adopted Mann–Whitney–Wilcoxon tests because the dependent variables were discrete (15-min intervals) and not normally distributed (Shapiro–Wilk normality tests, *P* < 0.001). To compare mating behavior between mating order (primary or secondary donor) within a reciprocating pair, we used paired Wilcoxon rank sum tests due to the same rationale as above (Shapiro–Wilk normality tests, *P* < 0.001).

To compare female reproductive output between the different ages and sizes, we used type III ANCOVAs, with age (young or old) and size (large or small) as fixed factors. We examined 4 dependent variables: total dry weight of egg masses, total number of eggs and egg masses, and egg size. As egg size, we used average egg area of all the egg masses laid by an individual. For total number of egg masses, we used a GLM with Poisson distribution. We also applied a type III multivariate ANCOVA to all measurements, providing the same independent variables.

To compare sex allocation between ages and sizes, we used type III ANCOVAs with similar settings as above but with whole-body weight (excluding target organs) as a covariate. We obtained dry weights of prostate glands, seminal vesicles, and albumen glands as dependent variables, which were square root transformed to obtain a normal distribution. For albumen glands, we added egg-laying status as a fixed factor. Also, we entered the matrix of these 3 variables and the covaraite into a type III multivariate ANCOVA.

In the postcopulatory male reluctance experiment, we used a GLM with binomial distribution entering whether focal snails inseminated their partner first, with treatment (control or inseminated focals) as a fixed factor. Given the sex role choice experiment, we tested whether the body size of primary donors was smaller than the recipients’ by testing for size differences within each pair using a paired *t*-test. All statistical analyses were conducted in R 3.0.1 (R Core Development Team 2013). For multivariate analyses, we used the R package “car.”

## RESULTS

### Field survey

**Figure 1:**
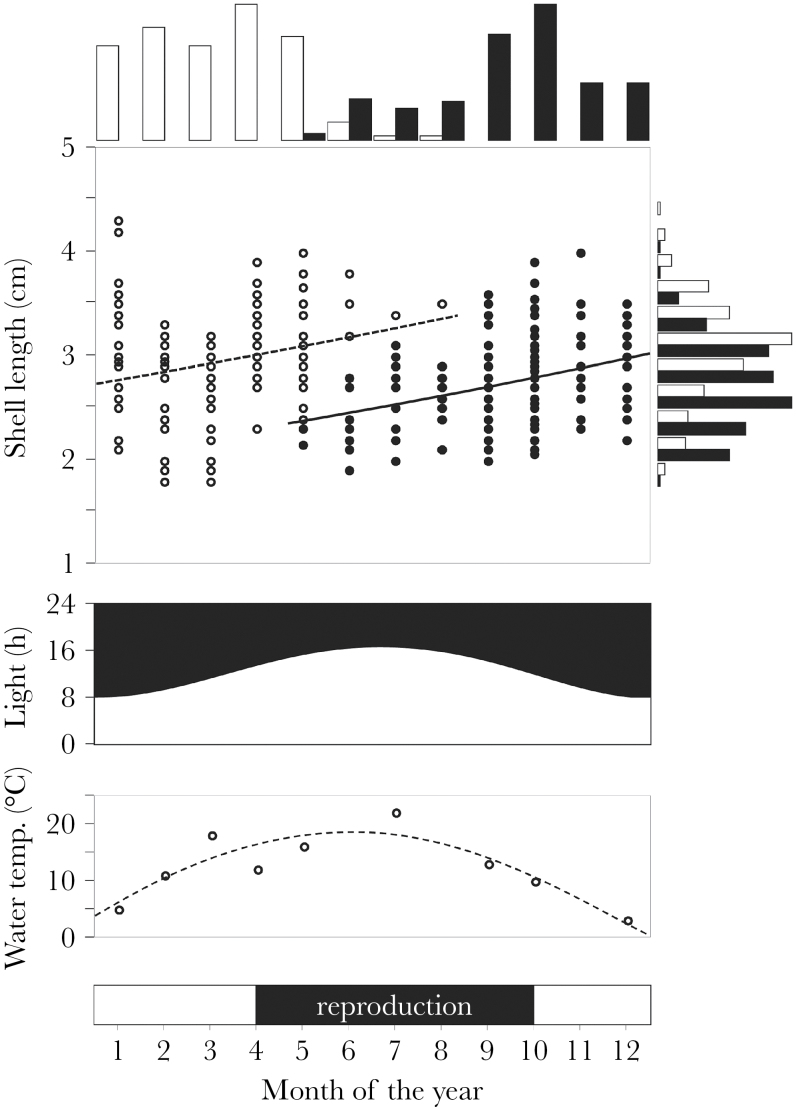
Field observation of a wild population of *Lymnaea stagnalis*. The top scatter plot depicts the shell length of the snails that were collected monthly over a 2-year period (data are depicted according to month of the year). Based on a K-means clustering on collection date and body size, the collected samples could be separated into 2 age cohorts (white and black circles). The top histograms represent the bimodal distribution of 2 age cohorts over the year (respectively, white and black), but their shell length distributions are similar, as shown by the histogram on the right side. As abiotic factors affecting their reproductive activity, the middle graph shows the daylight hours over the year and the bottom graph indicates the water temperature measured during the field sampling. The bar at the bottom of the figure indicates the period during which reproductive activities, that is, mating and egg laying, were observed (black).

Our field survey revealed a large variation in size in the studied population, so the range of shell length exceeded 5mm at any given month (Figure 1). Also, the collected individuals were clearly separated into 2 age cohorts, with a bimodal distribution of body size over time (Figure 1): one that newly emerged in spring and the other that originated from the preceding year. Estimates of the Von Bertalanffy growth rate show that these 2 cohorts do not grow differently as the 95% confidence intervals (CIs) overlap (Figure 1; mean ± 95% CI: open circles = 0.046±0.035–0.104; closed circles = 0.030±0.015–0.0046 per month).

There was seasonal variation in the proportion of infected individuals, which was lower during the winter compared with the rest of the year ($\chi_{11}^{2}=38.50$, *P* < 0.001, *N* = 487). Additionally, we observed reproductive activity (e.g., mating pairs and egg laying) from April until October, suggesting that individuals from different generations could copulate with each other, particularly at the start of the breeding season. Therefore, it is reasonable to assume that copulating pairs in the wild can have 5-mm shell length difference while being the same age, but may also have over 1-month, up to 1-year, age difference and have the same size.

### Sex role choice experiment

**Figure 2:**
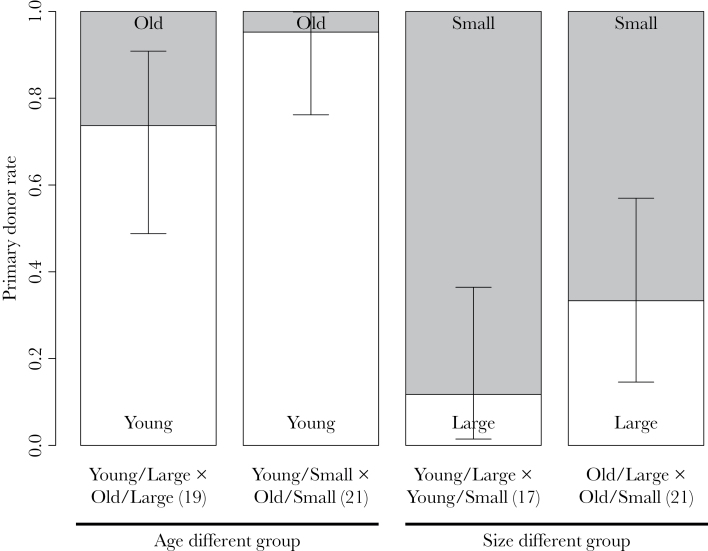
Mating role frequency in the sex role choice experiment. Primary donor rate is shown as proportion of individuals that inseminate first in a pair of snails with same size but different age (age-different group) and same age but different size (size-different group). The numbers in parentheses indicate sample size of each treatment, and error bars indicate 95% CIs.

**Figure 3:**
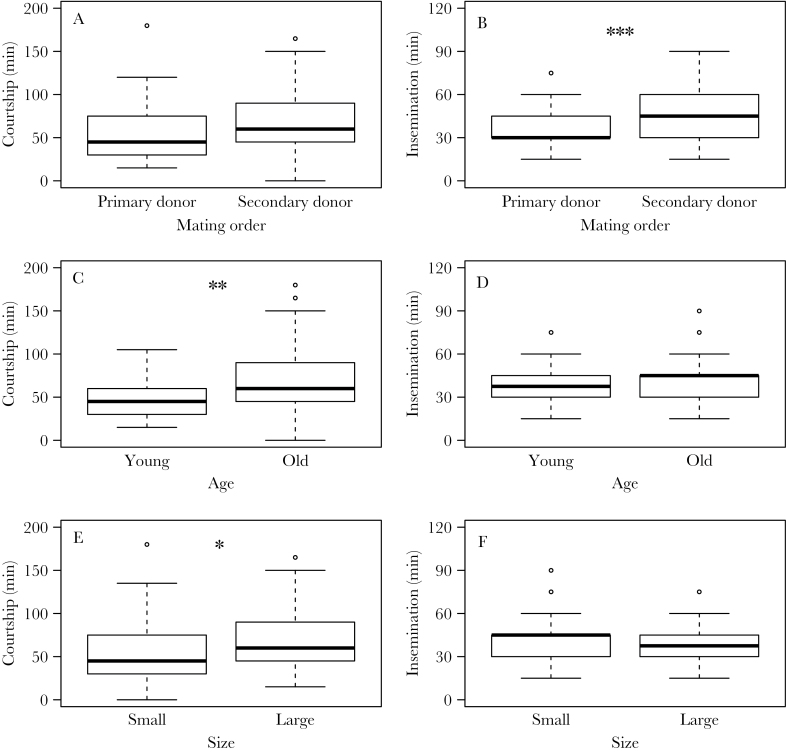
Mating behavior in the sex role choice experiment. Courtship and insemination duration are shown in comparison to mating order (A and B), age (C and D), and size (E and F). Because each pair includes primary and secondary donors, we used paired Wilcoxon rank sum tests. Asterisks indicate significant differences between groups (*P < 0.05; **P < 0.01; ***P < 0.001). The whiskers indicate minimum and maximum values, the box is for quartiles, and the thick line in the box stands for median. The open circles show outliners.

We observed 78 reciprocating pairs (2 age-different and 4 size-different pairs did not reciprocate). Young and small snails tend to be male first (binomial tests, 34 out of 40 cases in age-different treatment, *P* < 0.001; 29 out of 38 cases in size-different treatment, *P* = 0.002: Figure 2). Also in the age-different treatment, young and small snails were significantly more likely to be primary donors (GLM: size, $\chi_{1}^{2}=3.88$, *P* = 0.049: Figure 2), but this was not the case in the size-different treatment (GLM: age, $\chi_{1}^{2}=2.55$, *P* = 0.110). The latter implies an interactive effect of age and size. Mating behavior also differed between mating order, age, and size (Figure 3). Primary donors had significantly shorter insemination durations than secondary ones (paired Wilcoxon test: *V* = 425.5, *P* < 0.001: Figure 3B), but they did not differ in courtship durations (paired Wilcoxon test: *V* = 668, *P* = 0.067: Figure 3A). Both old and large snails took longer for courtship (Mann–Whitney test: age, *W* = 2206, *P* = 0.003; size, *W* = 3584, *P* = 0.050; Figure 3C,E), but they did not differ in insemination duration (Mann–Whitney test: age, *W* = 2866, *P* = 0.536; size, *W* = 2880, *P* = 0.557; Figure 3D,F) compared with either young or small snails.

### Female reproductive output

Contrasting with their clear behavioral choice for sex roles, female reproductive output neither differed between age nor size, regardless of whether it was measured as total dry weight of egg masses, total number of egg masses and eggs, or egg size (*N* = 69; Supplementary Figure S1 and Table S1). Only the multivariate test for overall female reproductive output showed significant difference between size classes (MANOVA: size, Pillai = 3.50, *P* = 0.013: Supplementary Table S1). Though this probably implies the general trend that larger snails have higher egg production, the potential benefits of inseminating large snails is small and strongly contrasts with their behavioral pattern.

### Sex allocation

**Figure 4:**
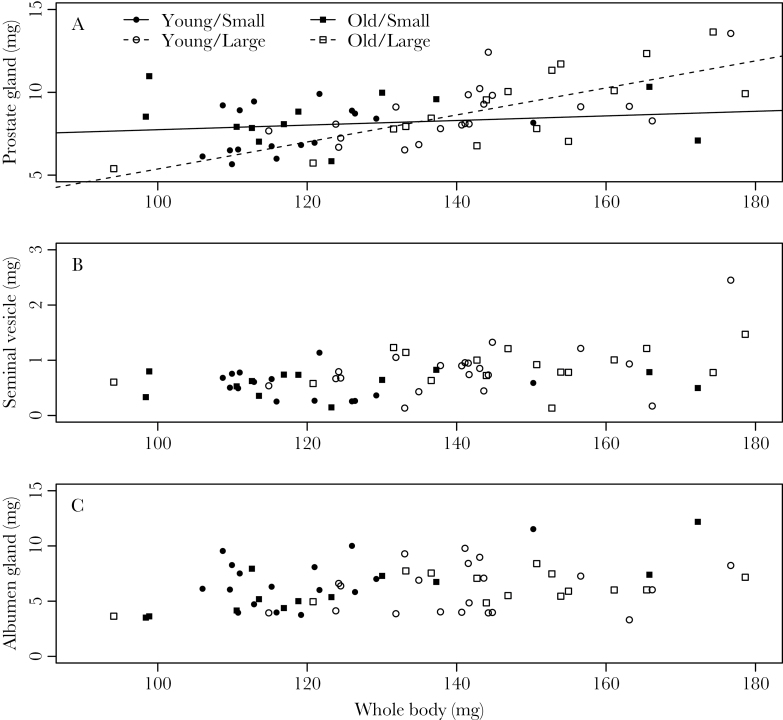
Sex allocation proxies of *Lymnaea stagnalis* of different ages and sizes. Dry weight of prostate glands, seminal vesicles (male investment: A and B), albumen glands (female investment: C), and whole-body weight are represented. The lines in the first figure represent significant regressions with whole-body weight. A closed symbol indicates a small individual and an open symbol a large one. The 2 types of symbols, square and circle, show age difference, although we did not detect any difference between ages (see Results).

In addition, we found that small snails invest more in their male function. Based on a multivariate test, body size seemed to have an effect on their sex allocation (Supplementary Table S2). Consistently, prostate gland weight (male organ) increased with body size (ANCOVA: size, *F*_1,56_ = 5.28, *P* = 0.025; whole body, *F*_1,56_ = 22.88, *P* < 0.001; Figure 4A and Supplementary Table S2), but not with age (ANCOVA: *F*_1,56_ = 1.89, *P* = 0.175). It is noteworthy that small snails had relatively larger prostate glands, given their body size (ANCOVA: size × whole body, *F*_1,56_ = 9.82, *P* = 0.003: Figure 4A). On the other hand, seminal vesicles only differed between size treatments (ANCOVA: size, *F*_1,54_ = 10.14, *P* = 0.002; Figure 4B) but did not correlate with other factors (Supplementary Table S2). Last, as a female organ, albumen gland weight depended primarily on egg-laying status (ANCOVA: *F*_1,47_ = 23.86, *P* < 0.001) and whole-body weight (ANCOVA: *F*_1,47_ = 18.69, *P* < 0.001; Figure 4C and Supplementary Table S2). Although there is an interaction between age and size (ANCOVA: *F*_1,47_ = 5.76, *P* = 0.020), the effect of age and size did not affect albumen gland weight (ANCOVA: age, *F*_1,47_ = 0.17, *P* = 0.683; size, *F*_1,47_ = 0.58, *P* = 0.452). Therefore, small snails invested relatively more in their male function, whereas large and/or old snails did not clearly invest more in their female function.

### Postcopulatory male reluctance experiment

In the postcopulatory male reluctance experiment, we did not detect any reduction in male mating motivation after being inseminated. We obtained 44 reciprocally mated pairs (1 pair in the inseminated group did not mate reciprocally). Eight of the 14 recently inseminated focal snails mated as primary donors, and 18 out of 30 control focals were primary donors (GLM: $\chi_{1}^{2}=0.01$, *P* = 0.861). The primary focal donors did not differ between treatments (control or inseminated) in insemination duration or in body size (*P* > 0.4). Although these snails were the same age, there was a slight difference in size within the pairs (0.49±0.38mm) because they were randomly paired with respect to body size. Such a difference in size was too small to detect a possible effect of size on being primary donor (paired *t*-test: *t*_43_ = −1.26, *P* = 0.214).

## DISCUSSION

When 2 fully male-motivated *L. stagnalis* interact to copulate, both age and size influence sex role decisions, whereas the recent receipt of ejaculate has no bearing on this decision. Small snails are predominantly primary donors, particularly when they are young (Figure 2). Although age is probably the major factor influencing sex role decisions (Hermann et al. 2009), our results are the first to experimentally disentangle and evaluate the effects of both age and size in this species. Furthermore, we show that such an age- and size-dependent sex role choice could occur in the wild, given the overlapping generations and considerable variation in body size (Figure 1). In general, this study emphasizes the unexplored link between age and size on sex role decisions in hermaphrodites (Table 1).

The observed mating pattern, where young and/or small snails inseminate first, could arise from either a harmonious mating agreement or a sexual conflict (Arnqvist 1992; Arnqvist and Rowe 2005). If the partners have complementary mating interests, our results would fit the size-advantage model or size-depending sex allocation strategies (Ghiselin 1969; Charnov 1982; Angeloni et al. 2002). In this scenario, large and/or old snails invest more in their female function and show higher female reproductive success, so they are willing to mate as females. In contrast, small and/or young snails invest more in their male function and are therefore more willing to mate as males. Consistently, large individuals usually produce more eggs (*L. stagnalis*, Koene et al. 2007; Hermann et al. 2009; Hoffer et al. 2012; other hermaphroditic taxa, Anthes and Michiels 2007; Jordaens et al. 2007). Furthermore, our experiment demonstrated that small snails have relatively large prostate glands (Hermann et al. 2009; Figure 4), which hints at their male-biased sex allocation, if one assumes that prostate gland weight is indicative of male sex allocation (Schärer 2009). However, within the range of our experimental setup (1 month and 5-mm shell length difference), we did not detect a clearly higher egg production in large or old snails, which is required for a harmonious mating agreement between mating partners with different ages and sizes. Although *L. stagnalis* can live for more than a year in captivity (Janse et al. 1989; Hermann et al. 2009), we refer to 4-month-old snails as old for the sake of simplicity. Nonetheless, given the age and size differences used in our experimental setup, it is unlikely that, at this life stage, the 2 factors are guiding a harmonious agreement between 2 mates.

Alternatively, we can interpret our results to illustrate that the mating interests of the mating partners are conflicting. In accordance with Van Duivenboden and Ter Maat (1985), 2 previously isolated individuals in a pair were equally and highly motivated to mate as male, implying a conflicting sexual interest. Furthermore, any snail would be motivated to inseminate first because primary donors transfer approximately double the amount of sperm, giving higher paternity success, than secondary donors (Nakadera et al. 2014). To inseminate a mating partner, however, they have to court and position themselves on the shell of the partner (Jarne et al. 2010; Koene 2010). Importantly, female-acting snails are not always passive and cooperative, which is well documented in other freshwater snails, *Physa* spp. (e.g., shell swinging, genital biting, DeWitt 1996; McCarthy 2004; Facon et al. 2006, 2007; McCarthy and Sih 2008). To accomplish this rather difficult task, high locomotory or adhesive ability can be pivotal. Young and small snails may have advantages to position themselves and maintain access to the female gonopore, supported by their shorter courtship duration (Figure 3C,E). Hence, the observed mating pattern can arise from behavioral interactions between 2 individuals with conflicting interests and is possibly settled by the locomotion or adhesive ability. Although the proximate mechanism (if any) to detect the age or size of conspecifics is largely unknown, our hypothesis proposes that locomotive ability alone may determine primary donors and thus individuals may not need to perceive their partners’ age or size.

In this experiment, we disentangled age and size effects on sex role decision using a two-by-two factorial design, though some confounding factors still remain. For instance, mating experience could affect their mating role decision (Hermann et al. 2009). Mating experience most likely correlates with age and indirectly with size. Hypothetically, Old/Large well-experienced snails could readily inseminate or reject a mate, but our behavioral data do not support this scenario. Nonetheless, the influence of mating experience on sex role decision is very intriguing to examine in future.

Our postcopulatory male reluctance experiment demonstrated that recent receipt of seminal fluid does not decrease male mating motivation, despite their reduced male performance. This may be due to the fact that our individuals were isolated, whereby mating in the male role is less costly, given their fully replenished prostate glands and seminal vesicles (for seminal fluid and sperm, respectively). This is also supported by the fact that the content of a full prostate gland can be enough for multiple matings, up to 3 inseminations (Koene and Ter Maat 2007; Koene et al. 2009). Alternatively, male-acting snails could gain more benefits by using their stored ejaculate components in order to prepare fresh ones. These 2 lines of thought can explain why recently inseminated snails are still motivated to mate as males compared with noninseminated controls. Moreover, this lack of reluctance after being inseminated suggests that it is not a target trait for manipulation via seminal fluid proteins. Thus, receipt of seminal fluid seems not to change male mating motivation in *L. stagnalis*.

In sum, we found that both age and size influence sex role decisions in *L. stagnalis* and that age seems to have the stronger influence. This identified effect of age and size would expand our understanding of sex role decisions in hermaphrodites (Hermann et al. 2009), which often give inconsistent conclusions (Table 1). Although recently inseminated *L. stagnalis* show a reduction in their male performance (sperm transfer, paternity success, Nakadera et al. 2014), they do not show any reluctance to inseminate a mate. The clear mating pattern (young and/or small snails inseminate first) does not support the size-advantage model, with no explicit evidence supporting that old or large individuals are better females with the tested range of age and size differences. It may still be there for larger differences in age (>1 month) or size (> 5mm in shell length), as found in the field population. Also, it is worth while to note that the snails may be choosing their sex roles based on traits different from those we have currently quantified. Collectively, our results suggest the importance of sexual conflict in precopulatory processes of *L. stagnalis*; yet, further investigation is required for the proximate mechanism and impact on overall reproductive success.

## SUPPLEMENTARY MATERIAL

Supplementary material can be found at http://www.beheco.oxfordjournals.org/

## FUNDING

This research was supported by the Research Council for Earth and Life Sciences (ALW) with financial aid from the Netherlands Organization for Scientific Research (NWO) via grants to J.M.K. (819.01.007) and a PhD grant from the Japan Student Services Organization (JASSO) to Y.N. This publication was supported by an Open Access grant of NWO.

## Supplementary Material

Supplementary Data
