# Supplementary material for: Effects of age, size, and mating history on sex role decision of a simultaneous hermaphrodite
Source: Behav Ecol. 2014 Oct 17;26(1):232–41. doi: 10.1093/beheco/aru184 (PMC4309981; doi:10.1093/beheco/aru184)
Supplement: Supplementary Data [file supp_26_1_232__index.html]

Effects of age, size, and mating history on sex role decision of a simultaneous hermaphrodite — Effects of age, size, and mating history on sex role decision of a simultaneous hermaphrodite — Supplementary Data 

# Effects of age, size, and mating history on sex role decision of a simultaneous hermaphrodite

## Supplementary Data

Data files

**Files in this Data Supplement:**

- Supplementary Data - Supplementary Data
