# Supplementary material for: Effects of age, size, and mating history on sex role decision of a simultaneous hermaphrodite
Source: Behav Ecol. 2014 Oct 17;26(1):232–41. doi: 10.1093/beheco/aru184 (PMC4309981; doi:10.1093/beheco/aru184)
Supplement: Supplementary Data [file supp_aru184_Supplemental_information.docx]

Supplemental information

Figure S1. Female reproductive output.The whiskers indicate min and max values, the box is quartiles, and the thick line in the box stands for median. The open cicles show outliners. Abbreviation: Nr = number of.

Table S1. Statistical results of female reproductive output. The combined matrix of four variables was used as female reproductive output for multivariate MANCOVA. Significant p-value is indicated in italics. Abbreviations: Nr = number of; d.f. = degree of freedom; LR = likelihood ratio.

Table S2. Statistical results of sex allocation test. As a variable of sex allocation, we used the combined matrix of prostate gland, seminal vesicle and albumen gland weights. Significant p-values are indicated in italics.

Figure S1.

Table S1

|  |  | Egg mass | | Nr eggs | | Nr egg mass | | Egg size | | Female reproductive output | |
| --- | --- | --- | --- | --- | --- | --- | --- | --- | --- | --- | --- |
| Model | d.f. | F value | P value | F value | P value | LR | P value | F value | P value | Pillai | P value |
| Age | 1 | 0.01 | 0.934 | <0.01 | 0.953 | 0.32 | 0.574 | 0.06 | 0.804 | 0.90 | 0.498 |
| Size | 1 | 0.01 | 0.906 | 0.21 | 0.648 | 0.56 | 0.453 | 0.26 | 0.613 | 3.50 | *0.013* |
| Age × Size | 1 | 0.68 | 0.412 | 0.04 | 0.836 | <0.01 | 0.996 | 0.11 | 0.743 | 1.90 | 0.120 |

Table S2.

|  |  | Prostate gland* | | Seminal vesicle* | | Albumen gland* | | Sex allocation | |
| --- | --- | --- | --- | --- | --- | --- | --- | --- | --- |
| Model | d.f. | F value | P value | F value | P value | F value | P value | Pillai | P value |
| Age | 1 | 1.89 | 0.175 | 0.40 | 0.531 | 0.17 | 0.683 | 0.01 | 0.939 |
| Size | 1 | 5.28 | *0.025* | 10.14 | *0.002* | 0.58 | 0.452 | 0.13 | 0.065 |
| Whole body (WB) | 1 | 22.88 | *<0.001* | 2.76 | 0.103 | 18.69 | *<0.001* | 0.36 | *<0.001* |
| Age × Size | 1 | 0.51 | 0.480 | 0.01 | 0.918 | 5.76 | *0.020* | 0.06 | 0.408 |
| Age × WB | 1 | 0.57 | 0.454 | 0.30 | 0.588 | 0.62 | 0.436 | 0.01 | 0.917 |
| Size × WB | 1 | 9.82 | *0.003* | 1.48 | 0.230 | 2.48 | 0.122 | 0.15 | *0.047* |
| Age × Size × WB | 1 | 1.67 | 0.202 | 1.47 | 0.231 | 0.37 | 0.547 | 0.05 | 0.422 |
| Egg laying | 1 |  |  |  |  | 23.86 | *<0.001* |  |  |
| Age × Egg laying | 1 |  |  |  |  | 3.87 | 0.055 |  |  |
| Size × Egg laying | 1 |  |  |  |  | 3.71 | 0.060 |  |  |
| WB × Egg laying | 1 |  |  |  |  | 0.99 | 0.325 |  |  |
| Age × Size × Egg laying | 1 |  |  |  |  | <0.01 | 0.952 |  |  |
| Age × WB × Egg laying | 1 |  |  |  |  | 3.65 | 0.062 |  |  |
| Size × WB × Egg laying | 1 |  |  |  |  | 0.14 | 0.706 |  |  |
| * square root transformed |  |  |  |  |  |  |  |  |  |
